# Four new species of *Pyropia* (Bangiales, Rhodophyta) from the west coast of North America: the *Pyropia lanceolata* species complex updated

**DOI:** 10.3897/phytokeys.52.5009

**Published:** 2015-06-25

**Authors:** Sandra C. Lindstrom, Jeffery R. Hughey, Luis E. Aguilar Rosas

**Affiliations:** 1 Department of Botany and Beaty Biodiversity Museum (Biodiversity Research Centre), #3529 – 6270 University Blvd, University of British Columbia, Vancouver, BC, Canada V6T 1Z4; 2 Division of Science and Mathematics, Hartnell College, 411 Central Ave., Salinas, CA 93901, U.S.A.; 3 Instituto de Investigaciones Oceanológicas, Universidad Autónoma de Baja California, A.P. 453, Ensenada, Baja California 22830, México

**Keywords:** Bangiales, British Columbia, California, new species, northeast Pacific, *Pyropia lanceolata* species complex, *Pyropia nereocystis*, *rbc*L gene

## Abstract

Recent molecular studies indicate that the *Pyropia lanceolata* species complex on the west coast of North America is more speciose than previously thought. Based on extensive *rbc*L gene sequencing of representative specimens we recognize seven species in the complex, three of which are newly described: *Pyropia montereyensis* **sp. nov.**, *Pyropia columbiensis* **sp. nov.**, and *Pyropia protolanceolata* **sp. nov.** The new species are all lanceolate, at least when young, and occur in the upper mid to high intertidal zone primarily in winter and early spring. *Pyropia montereyensis* and *Pyropia columbiensis* are sister taxa that are distributed south and north of Cape Mendocino, respectively, and both occur slightly lower on the shore than *Pyropia lanceolata* or *Pyropia pseudolanceolata*. *Pyropia protolanceolata* is known thus far only from Morro Rock and the Monterey Peninsula, California; it occurs basally to the other species in the complex in the molecular phylogeny. A fourth newly described species, *Pyropia bajacaliforniensis* **sp. nov.**, is more closely related to *Pyropia nereocystis* than to species in this complex proper. It is a thin species with undulate margins known only from Moss Landing, Monterey Bay, California, and northern Baja California; it also occurs in the high intertidal in spring. *Porphyra mumfordii*, a high intertidal winter species that has frequently been confused with species in the *Pyropia lanceolata* complex, has now been confirmed to occur from Calvert Island, British Columbia, to Pescadero State Park, California.

## Introduction

The foliose Bangiales are one of the best-studied groups of marine red algae occurring on the west coast of North America. The first two species to be named from the region were two of the most common, *Porphyra perforata* J. Agardh (1883) and *Porphyra nereocystis* C.L. Anderson (Blankinship & Keeler, 1892). Hus (1900, 1902) summarized knowledge of the genus on the Pacific Coast, recognizing eleven species and describing three new forms of *Porphyra* C. Agardh, the genus to which all foliose Bangiales belonged at the time. One of those new forms, Porphyra perforata f. lanceolata Setchell & Hus in Hus (1900), was erected to accommodate lanceolate forms that were dioecious; this taxon was later raised to specific status in Smith and Hollenberg (1943: 213), who also added two more species of *Porphyra* to the flora. It was Krishnamurthy (1972) who significantly revised the genus in the region and added seven new species, mostly from Washington State. A summary of knowledge at the time was provided by Conway et al. (1975), with detailed descriptions of Pacific Northwest species of *Porphyra* with emphasis on British Columbia and Washington State; their work was updated by Garbary et al. (1981).

Studies up to then mostly utilized thallus morphology and the pattern of reproductive cell disposition and division as defining features for species. Mumford and Cole (1977) added chromosome numbers as a useful feature, and Lindstrom and Cole (1990, 1992a, b, c) and Lindstrom (1993) utilized isozymes in addition to morphology, chromosome numbers, biogeography and habitat as characters for separating and recognizing even more species.

The taxonomy of foliose Bangiales entered a new phase with the application of DNA sequencing methods. Lindstrom and Fredericq (2003) sequenced the chloroplast *rbc*L gene of many West Coast species, and Lindstrom (2008) included numerous additional specimens, indicating the need to describe even more species, as did Kucera and Saunders (2012) utilizing the mitochondrial 5´end of the *COI* gene. Sequencing also indicated that a wholesale revision of the order was needed (first suggested by Oliveira et al. 1995). This led to a revision of the genera of foliose Bangiales by Sutherland et al. (2011), redefining, resurrecting or creating eight genera of bladed Bangiales. Among these eight genera, four (*Boreophyllum* S.C. Lindstrom, *Fuscifolium* S.C. Lindstrom, *Porphyra* and *Pyropia* J. Agardh) occur on the west coast of North America, and among these *Pyropia* is by far the most speciose.

The resurrected genus *Pyropia* contains a number of clades that are resolved with substantial support, and many of these clades are biogeographically circumscribed (Sutherland et al. 2011). One such clade is the northeast Pacific *Pyropia lanceolata*–*Pyropia pseudolanceolata* complex, first identified as such by Lindstrom and Cole (1992b), who recognized that a number of species were confused under these names. Members of this clade, like other species of *Pyropia*, have monostromatic blades. As resolved by Sutherland et al. (2011), this clade contains *Pyropia* sp. 480, *Pyropia pseudolanceolata* (V. Krishnamurthy) S.C. Lindstrom, *Pyropia hiberna* (S.C. Lindstrom & K.M. Cole) S.C. Lindstrom, *Pyropia fallax* (S.C. Lindstrom & K.M. Cole) S.C. Lindstrom, *Pyropia conwayae* (S.C. Lindstrom & K.M. Cole) S.C. Lindstrom, and *Pyropia* sp. 485, indicating that at least two species are as yet undescribed and suggesting uncertainty over the identity of *Pyropia lanceolata* (Setchell & Hus) S.C. Lindstrom.

In the present study, we analyzed *rbc*L and 18S rRNA (SSU) gene sequences from recently collected specimens belonging to this clade from the west coast of North America extending from Baja California to Alaska. We also include the closely related northeast Pacific species *Pyropia nereocystis* and *Pyropia kanakaensis* (Mumford) S.C. Lindstrom (Lindstrom 2008, Sutherland et al. 2011), and we analyzed short DNA sequences from the type sheets of *Pyropia lanceolata* and *Pyropia hiberna* to resolve their relationship, and to determine whether any of the undescribed species could be the same as one of these species. These new data support the recognition of at least four additional species. Below we discuss these species, their relationships to each other, and the characters that distinguish them.

## Materials and methods

Specimens were collected by the authors or by those named in the Acknowledgments (Table 1, Suppl. material 1). Collections were made along the west coast of North America from Baja California, Mexico, to the western tip of the Aleutian Islands, Alaska, between 1992 and 2014. Upon collection, the specimens were damp-dried and then desiccated in silica gel. Pieces or separate specimens were pressed to make herbarium vouchers, which are deposited in UBC or UC. Silica-gel dried specimens were returned to the lab, where they were extracted following the CTAB protocol as implemented by Lindstrom and Fredericq (2003). PCR amplification and sequencing of the *rbc*L gene was carried out as described in Lindstrom (2008) except that KitoF1 (5’ atgtctcaatccgtagaatca 3’) was used as the forward primer rather than F57. DNA from type material of *Pyropia lanceolata* and *Pyropia hiberna* was extracted, amplified and sequenced following the protocol described in Lindstrom et al. (2011), except for using 3X the primer concentration used previously. The type fragments were extracted in a separate laboratory (Hartnell College) and processed employing the precautionary steps proposed by Hughey and Gabrielson (2012). For amplification of type material, primers F625 (5'CTCACAACCATTTATGCGTTGG 3’) and R900 (5'GCGAGAATAAGTTGAGTTACCTG 3’) were cycled together.

**Table 1.** Specimens for which the *rbc*L gene was sequenced in this study and used in the phylogenetic analyses. All herbarium vouchers are deposited in UBC unless noted otherwise. Numbers indicate the total number of specimens with the identical sequence (see Suppl. material 1). Specimens in brackets were included in initial analyses but excluded from the analysis shown in Fig. 1. Specimen P814 in Fig. 1 represents a combination of P814 and P827 (Calvert I., BC, 27 May 2013, K. Hind, SCL 15332, KP904063) to provide a more complete sequence.

Extract	Collection site	Collection date	Collector	Collection no.	GenBank no.	Specimens with identical sequence
*Pyropia fallax*						
[P172	Clover Pt, BC, Canada	27 Apr 2002	S.C. Lindstrom	no voucher?	EU223056	n=5]
P191	Harling Pt, BC, Canada	25 Apr 2005	S.C. Lindstrom	SCL 12565	EU223057	unique
[P225	Akutan Bay, AK, USA	31 Jul 2004	S.C. Lindstrom	SCL 11611	EU223064	unique]
P525	Chichagof Hbr, AK, USA	04 Jun 2008	S.C. Lindstrom	SCL 13483	KP903917	unique
P544	Surveyor Bay, AK, USA	11 Jun 2008	S.C. Lindstrom	SCL 13709	KP903919	n=2
P557	Foster I., BC, Canada	27 May 2009	S.C. Lindstrom	SCL 14121	KP903922	n=23
P577	Hallo Bay, AK, USA	03 Jul 2009	M.R. Lindeberg	UBC A89044	KP903923	n=10
[P815	Calvert I., BC, Canada	25 May 2013	S.C. Lindstrom	SCL 15293	KP903936	n=2]
P820	Calvert I., BC, Canada	26 May 2013	S.C. Lindstrom	SCL 15304	KP903940	n=2
P851	Calvert I., BC, Canada	18 Feb 2014	S.C. Lindstrom	SCL 15595	KP903948	n=17
*Pyropia conwayae*						
P430	French Beach, BC, Canada	12 Mar 2007	S.C. Lindstrom	SCL 13109	EU223044	n=2
[P494	Charleston, OR, USA	04 Apr 2008	S.C. Lindstrom	SCL 13303	KP903957	n=2]
P589	Camel Rock, CA, USA	14 Feb 2010	S.C. Lindstrom	SCL 14287	KP903961	n=13
*Pyropia montereyensis*						
P603	Fort Bragg, CA, USA	15 Feb 2010	S.C. Lindstrom	SCL 14311	KP903964	n=7
P645	N of San Simeon, CA, USA	18 Feb 2010	S.C. Lindstrom	SCL 14374	KP903967	n=4
P656	S of Ventura Beach, CA, USA	20 Feb 2010	S.C. Lindstrom	SCL 14392	KP903968	unique
P763	Spanish Bay, CA, USA	02 Feb 2012	J.R. Hughey	UBC A90632	KP903972	n=4
*Pyropia columbiensis*						
P491	Trinidad St. Beach, CA, USA	12 Apr 2008	F.J. Shaughnessy	Frank#1 in HSC	KP903982	n=2
P859	Calvert I., BC, Canada	18 Feb 2014	S.C. Lindstrom	SCL 15599	KP903999	n=20
*Pyropia lanceolata*						
[P584	Trinidad boat launch ramp, CA, USA	14 Feb 2010	S.C. Lindstrom	SCL 14276	KP904008	unique]
P612	Van Damme St. Park, CA, USA	16 Feb 2010	S.C. Lindstrom	SCL 14321	KP904024	unique
P625	Bodega Marine Lab, CA, USA	16 Feb 2010	S.C. Lindstrom	SCL 14341	KP904029	unique
P638	Pescadero St. Park, CA, USA	17 Feb 2010	S.C. Lindstrom	SCL 14365	KP904038	n=39
P641	Pacific Grove, CA, USA	17 Feb 2010	S.C. Lindstrom	SCL 14369	KP904039	n=2
*Pyropia pseudolanceolata*						
[P332	Chaichei Islets, AK, USA	20 Apr 1995	S.C. Lindstrom	SCL 9104	KP904049	unique]
P351	Dundas I., BC, Canada	19 Apr 2007	S.C. Lindstrom	SCL 13136	EU223163	n=7
P411	Sedanka Pt, AK, USA	03 Jun 2005	S.C. Lindstrom	SCL 12137	EU223165	unique
P488	Sunset Beach, OR, USA	06 Apr 2008	S.C. Lindstrom	SCL 13311	KP904052	n=3
P537	Alaid I., AK, USA	07 Jun 2008	S.C. Lindstrom	SCL 13630	KP904056	n=28
*Pyropia protolanceolata*						
P480	Spanish Bay, CA, USA	01 Jan 2008	P.W. Gabrielson	PWG 1604	KP904005	same as P797
					KP903902 (SSU)	
P767	Morro Rock, CA, USA	04 Apr 2012	J.R. Hughey	UBC A90634	KP904006	same as P480
					KP903909 (SSU)	
*Pyropia kanakaensis*						
Pkan	Kanaka Bay, WA, USA	undated	M.J. Wynne	MICH	AF452431	unique
P132	Baker Beach, CA, USA	25 May 2002	S.C. Lindstrom	SCL 11409	EU223098	n=3
P222	Olympic Pen., WA, USA	31 May 2003	S.C. Lindstrom	SCL 10932	EU223099	unique
*Pyropia bajacaliforniensis*						
P766	Moss Beach Jetty, CA, USA	30 Apr 2012	J.R. Hughey	no voucher?	KP904065	same as *Pyropia* sp. MIG
*Pyropia* sp. MIG	Faro de San Miguel, BC, Mexico			WELT A024422	HQ687536	same as P766
*Pyropia* sp. FAL	Saldamando, BC, Mexico	21 May 2002	L.E. Aguilar Rosas & R. Aguilar Rosas	WELT A024418	HQ687535	unique
*Pyropia* sp.						
s/n	San Carlos Beach Park, CA, USA	05 Jan 2015	J.R. Hughey	UC 1966781	KP876025	unique
*Pyropia nereocystis*						
P320	Passage I., AK, USA	30 Jun 2003	M.R. Lindeberg	SCL 11215	EU223116	unique
P814	Calvert I., BC, Canada	24 May 2013	S.C. Lindstrom	SCL 15280	KP904062	n=6

Sequences of the *rbc*L gene of *Pyropia* sp. FAL from Playa Saldamando, Baja California, Mexico, HQ687535, and *Pyropia* sp. MIG from Faro de San Miguel, Baja California, Mexico, HQ687536, were also included in the analyses because of their close relationship to *Pyropia kanakaensis* and *Pyropia nereocystis* (Sutherland et al. 2011) and because of the identity of *Pyropia* sp. MIG with one of our unknown specimens. We selected two specimens of *Pyropia* sp. (AB118586 and AB287965) as outgroups based on their close genetic identity to *Pyropia nereocystis* using the GenBank blastn algorithm (accessed 06 Sept 2014).

Sequences were aligned using BioEdit version 7.0.9.1 (Hall 1999). Maximum parsimony (MP) analysis was performed using PAUP* 4.0b10 (Swofford 2002) as implemented by Lindstrom and Fredericq (2003). Maximum likelihood (ML) was performed using RAxML 7.2.6 [as implemented on the T-rex website (http://www.trex.uqam.ca/index.php?action=raxml; Stamatakis 2006, Buc et al. 2012)], and data were partitioned by codon position. Bayesian phylogenetic analyses were performed on the Bio-Linux7 platform (Field et al. 2006) with MrBayes 3.2.1 (Huelsenbeck et al. 2001, Ronquist and Huelsenbeck 2003). We followed the MrBayes 3.2 manual, which recommends continuing analyses by increasing the number of generations until the average standard deviation of split frequencies drops below 0.01. All runs were performed using a sample frequency of 10 with two independent analyses. To calculate the Potential Scale Reduction Factor and posterior probabilities, the sump and sumt burn-in values were set to discard 25% of the samples.

## Results

**Figure 1.**
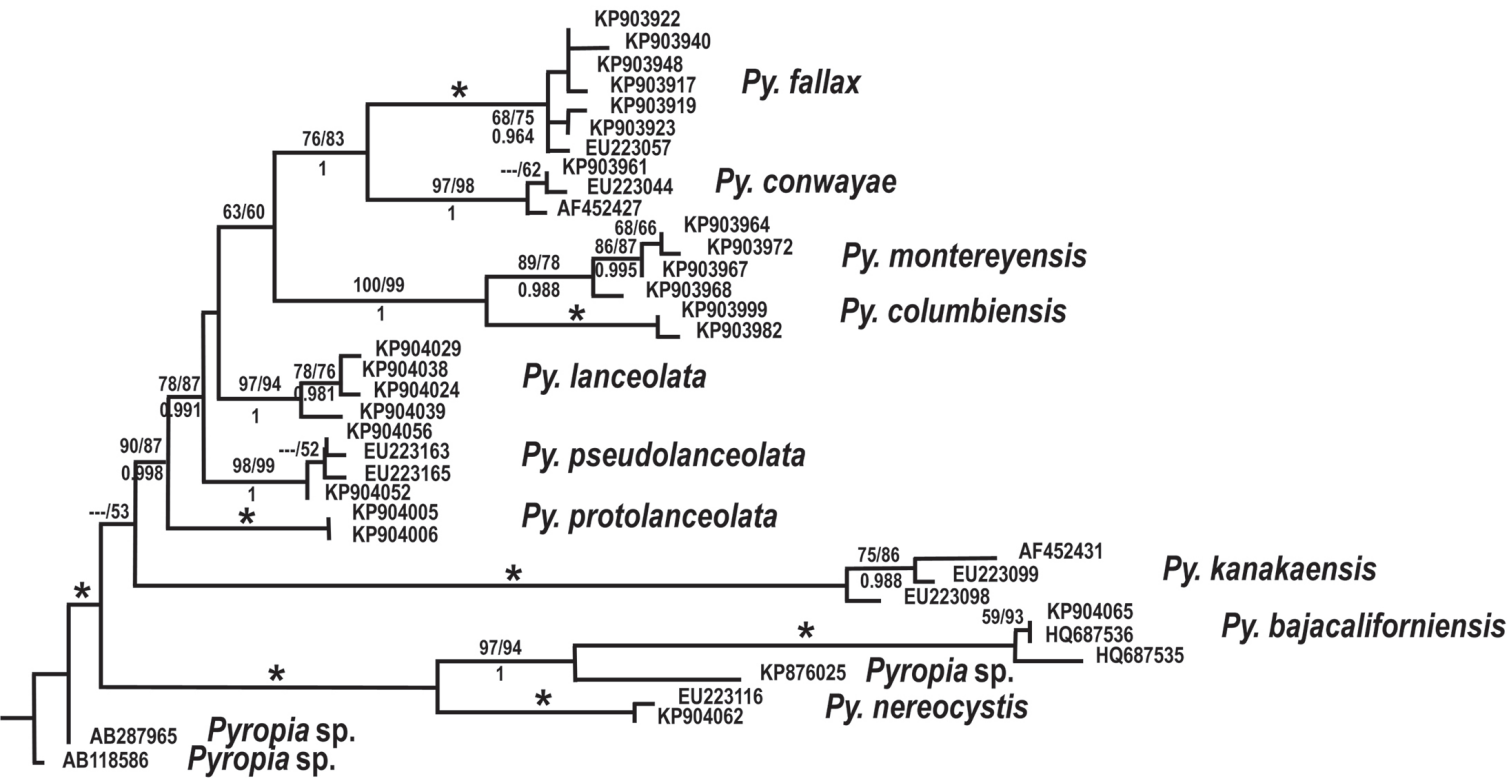
Maximum likelihood tree of the *Pyropia lanceolata* complex and close relatives. An asterisk indicates 100% bootstrap support in (left to right) maximum parsimony (nreps=10000) and maximum likelihood (nreps=1000), above the line, and a Bayesian probability of 1.0 below the line. Only bootstrap values >50 and Bayesian probabilities >0.900 are shown.

Thirty-seven *rbc*L gene sequences (Table 1) were included in the phylogenetic analyses that generated Fig. 1. Both MP and ML generated the same tree topology, as did Bayesian analysis. Several unique sequences were omitted from the analyses after it was determined that their omission did not alter the topology of the phylogenetic tree. In addition to these sequences, the Suppl. material 1 includes 186 additional specimens that were identical to those in Fig. 1. With AB118586 and AB287965 as outgroup species, three major clades are apparent, the *Pyropia nereocystis* clade, the *Pyropia kanakaensis* clade and the *Pyropia lanceolata* clade (formerly called the *Pyropia lanceolata*–*Pyropia pseudolanceolata* complex).

Within the *Pyropia lanceolata* clade, *Pyropia protolanceolata* diverges first. This species is sister to *Pyropia pseudolanceolata*, then *Pyropia lanceolata*, but this order of divergence is without support. The clade is terminated by two pairs of sister taxa, the closely related *Pyropia montereyensis* and *Pyropia columbiensis* species pair, and the somewhat more distantly related *Pyropia conwayae* and *Pyropia fallax* pair. Both of these species pairs represent a southern and northern species, as is also the case for *Pyropia lanceolata* and *Pyropia pseudolanceolata*. For the most closely related pair, *Pyropia montereyensis* and *Pyropia columbiensis*, the former has to date only been found south of Cape Mendocino whereas the latter has only been collected from Cape Mendocino north; thus these species do not appear to overlap in their distributions. In the case of *Pyropia conwayae* and *Pyropia fallax*, the species overlap in distribution between southern Vancouver Island and southern Oregon. Of these species pairs, the former pair is more constrained in its distribution, occurring only between southern California and central British Columbia whereas the latter pair extends from central California to at least the westernmost Aleutian Island. For *Pyropia lanceolata* and *Pyropia pseudolanceolata*, this older species pair shows an even wider area of overlap, between Sitka Sound, AK, and Crescent City, CA. All species in the *Pyropia lanceolata* clade occur on strongly supported branches, and all but *Pyropia protolanceolata* show some intraspecific variation (to 0.4%) in their *rbc*L sequences (only two specimens of *Pyropia protolanceolata* were sequenced due to the infrequency of collection). The nonoverlapping intraspecific versus interspecific divergence, also referred to as the ‘‘barcode gap’’, allows specimens to be assigned unambiguously to genetic clusters that constitute putative genetic species (Le Gall and Saunders 2010).

In the *Pyropia nereocystis* clade, *Pyropia nereocystis* is sister to two divergent species. *Pyropia* sp. has been collected several times in early winter from the uppermost intertidal on the Monterey Peninsula; it is the subject of a separate study and will be described there. *Pyropia bajacaliforniensis*, the other species, has been collected in late spring on the central California and northern Baja California coasts. The type specimen, described below, diverges from two other collections by 0.3% (4 base pairs); this level of divergence is within the typical species variation exhibited by the *rbc*L gene in foliose Bangiales of up to 0.4% (Nelson and Broom 2010, Mols-Mortensen et al. 2012) although levels up to 1% have been reported for a few species (Lindstrom 2008).

*Pyropia kanakaensis* terminates its own long branch, suggesting a long evolutionary history separate from its closest relatives. It also shows significant within species variation.

We also sequenced the 18S rRNA gene in representatives of these species (Table 1, Suppl. material 1) to complement the data in Sutherland et al. (2011). There was relatively little variation among species and little structure to the phylogenetic tree except for weak support for sibling relationships between *Pyropia nereocystis* and *Pyropia kanakaensis* and between *Pyropia lanceolata* and *Pyropia pseudolanceolata*.

Characters of the species in the *Pyropia lanceolata* clade are summarized in Table 2. Most specimens are lanceolate with slightly undulate margins. All are monostromatic with one chloroplast per cell although chloroplast division prior to cell division can give the appearance of cells being vegetatively diplastidial. Among the species, only *Pyropia fallax* is monoecious, with spermatangial patches or streaks among pale red zygotosporangia, which occur in submarginal patches, mottles, streaks or hieroglyphs. The remaining species are almost invariably dioecious, with spermatangia occurring along cream-colored margins and with the red zygotosporangia occurring along the margin and across the distal end of the thallus in patches usually intermixed with vegetative cells, giving the appearance of red hieroglyphs. All species occur on rock, often near sand.

**Table 2.** Comparison of morphological features of species in the *Pyropia lanceolata* clade.

Feature	*Pyropia fallax*	*Pyropia conwayae*	*Pyropia montereyensis*	*Pyropia columbiensis*	*Pyropia lanceolata*	*Pyropia pseudolanceolata*	*Pyropia protolanceolata*
Shape	Ovate to broadly lanceolate	Lanceolate	Lanceolate, occasionally oblanceolate	Lanceolate to somewhat ovate (rarely obovate)	Lanceolate	Lanceolate to ovate	Linear to lanceolate
Thickness	49–66 µm	53–113 µm	50–110 µm	50–115 µm	45–100 µm	65–150 µm	28–65 µm
Width (males)	to 5.0 cm	2.0–11.0 cm	to 2.3 cm	to 5.5 cm	1.2–1.5 cm	1.0–5.4 cm	to 1.2 cm
Length (males)	to 30 cm	to 83 cm	to 69 cm	to at least 31 cm	10–14 cm	to 31 cm	to 16 cm
Width (females)	same as males	4.0–8.2 cm	to 4.8 (10) cm	to 12 cm	1.0–3.5 cm	1.8–9.0 cm	not seen
Length (females)	same as males	to 40 cm	to 68 cm	to at least 28 cm	to 43 cm	to 34 cm	not seen
Color	Margin reddish, center greenish	Dark gray-green	Olive-green to grayish or brownish purple	Olive-green to grayish or brownish purple	Olive-green, brown (golden), or grayish purple	Olive-green to greenish gray or grayish purple	Dusky rose
Spermatangia	1–2 × 2–4 × 8	2–4 × 4 × 16	2–4 × 2–4 × 8–16	2–4 × 2–4 × 8	2–4 × 2–4 × 8	2–4 × 2–4 × 8	2 × 2 × 8
Zygotosporangia	in tiers of 4–8	2–4 × 2–4 × 2–4	2–4 × 2–4 × 4–8	2–4 × 2–4 × 2–4	2–4 × 2–4 × 4–8	2–4 × 2–4 × 4–8	not seen
Elevation	Mid to high intertidal	Mid intertidal	Mid to high intertidal	Mid to high intertidal	Upper mid to high intertidal	High intertidal	Very high intertidal
Phenology	Winter to late spring (mid summer in north)	Late winter to late spring	Winter to mid spring	Winter to early spring (rarely to mid summer)	Winter to early spring	Winter to early spring (mid summer in north)	Winter to early spring
Distribution	Attu I., AK, to southern OR	Tofino, BC, to Land’s End, San Francisco, CA	Fort Bragg to just south of Ventura, CA	Calvert I., BC, to Cape Mendocino, CA	Sitka Sound, AK, to Cambria, CA	Attu I., AK, to Crescent City, CA	Spanish Bay & Morro Bay, CA
Haploid chromosome number	2	2	unknown	unknown	3	3	unknown

Below we describe in detail the previously unnamed species in this clade, as well as a new species in the *Pyropia nereocystis* clade.

### Pyropia montereyensis

Taxon classificationPlantaeBangialesBangiaceae

S.C. Lindstrom & J.R. Hughey sp. nov.

Fig. 2

#### Description.

Thalli lanceolate and acuminate (occasionally oblanceolate) when young, becoming ovate to nearly orbiculate and often cleft when post-reproductive, base cuneate to strongly umbilicate when old; 50–75 mm thick when dried and young, 90–110 mm thick when old; males to at least 2.3 cm wide and 69 cm long; females to at least 4.8 cm wide and 68 cm long (although usually narrower; to 10 cm broad when old); color uniform throughout the thallus except for reproductive areas, olive green when fresh, drying to grayish or brownish purple. Thalli dioecious. Spermatangia in packets of 2–4 × 2–4 × 8–16. Zygotosporangia in packets of 2–4 × 2–4 × 4–8. Habitat: mid to high intertidal rock, usually associated with sand. Phenology: Winter to mid spring. Distinguished from other species of *Pyropia* by unique *rbc*L and 18S rRNA gene sequences.

#### Holotype.

Saxicolous in the upper intertidal on rocks partially buried in sand at the north end of Spanish Bay, Pacific Grove, California, USA (36°37.16'N 121°56.52'W), *Hughey*, 02 Feb 2014, *UC2050590*. GenBank sequence KP903972 (*rbc*L).

#### Isotypes.

*UBC A90632*.

#### Etymology.

This species is named for the biogeographic region in which it is found following the boundaries of Croom et al. (1995) more closely than those of Valentine (1966).

#### Distribution.

Fort Bragg to just south of Ventura Beach, California, USA.

We did not obtain an SSU sequence from type material of this species. The SSU sequence in GenBank (KP903907) for this species is from another Monterey Peninsula site: Carmel River State Beach.

**Figure 2.**
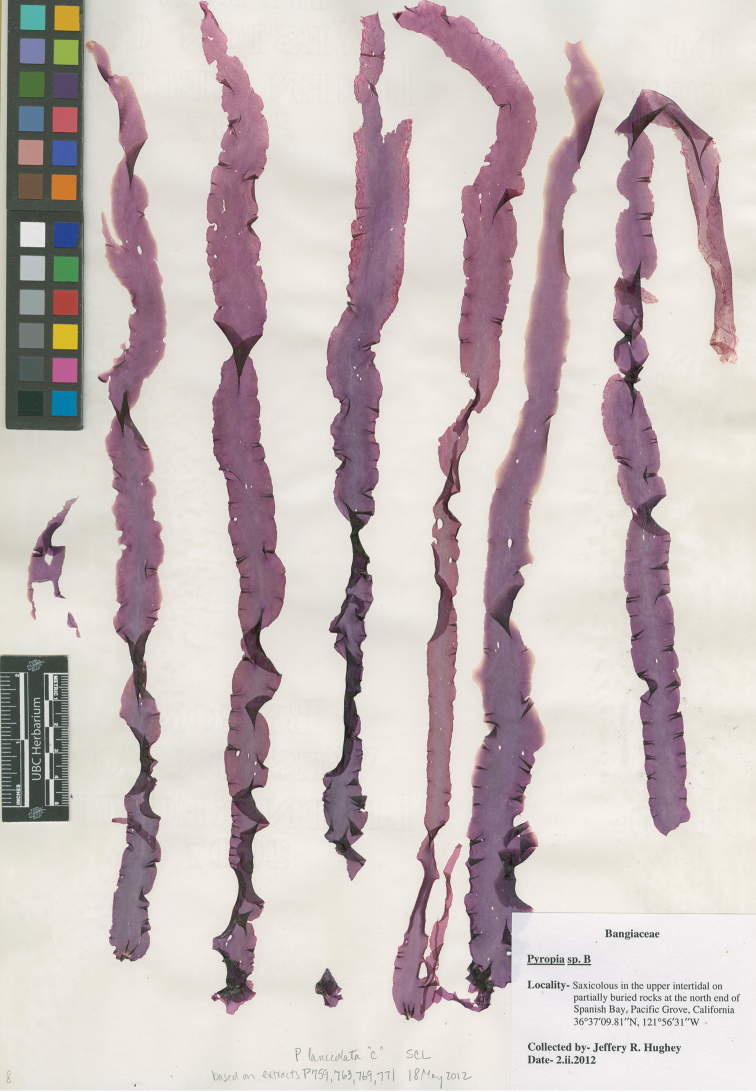
Holotype of *Pyropia montereyensis*. North end of Spanish Bay, Pacific Grove, California, USA, *Hughey*, 02 Feb 2014 (*UC2050590*).

### Pyropia columbiensis

Taxon classificationPlantaeBangialesBangiaceae

S.C. Lindstrom sp. nov.

Fig. 3

#### Description.

Thalli lanceolate when young, becoming somewhat ovate (rarely obovate) when mature; base cuneate, becoming umbilicate; 50–115 mm thick; males to at least 5.5 cm wide and more than 31 cm long; females to 12 cm wide and more than 28 cm long, but thalli mostly narrower; color uniform throughout the thallus except for reproductive areas, olive-green when fresh, drying to grayish or brownish purple. Thalli dioecious. Spermatangia in packets of 2–4 × 2–4 × 8. Mature zygotosporangia in packets of 2–4 × 2–4 × 2–4. Habitat: mid to high intertidal rock, usually associated with sand. Phenology: winter to early spring (a few thalli may persist as late as mid summer). Distinguished from other species of *Pyropia* by unique *rbc*L and 18S rRNA gene sequences.

#### Holotype.

Saxicolous in the upper mid intertidal on rocks partially buried in sand at the south end of West Beach, Calvert Island, British Columbia, Canada (51°39.14'N 128°08.42'W), *S.C. Lindstrom 15596*, 18 Feb 2014, *UBC A90636*. GenBank sequences KP903995, KP903996 (*rbc*L), KP903910 (SSU).

#### Isotypes.

SCL 15594 (*UC 2050591*), SCL 15599 (*UBC A90637*), SCL 15600 & 15601 (*UBC A90638*).

#### Etymology.

This species is named for the biogeographic region in which it is found, using the terminology of Valentine (1966), but with a modification of the boundaries to extend from Cape Mendocino, California, to the central coast of British Columbia. It also commemorates the centenary of the University of British Columbia herbarium, which was established in early 1916.

#### Distribution.

Calvert Island, British Columbia, Canada, to Cape Mendocino, California, USA.

*Pyropia montereyensis* and *Pyropia columbiensis* are essentially morphologically identical and represent the southern and northern species of a vicariant pair, respectively.

**Figure 3.**
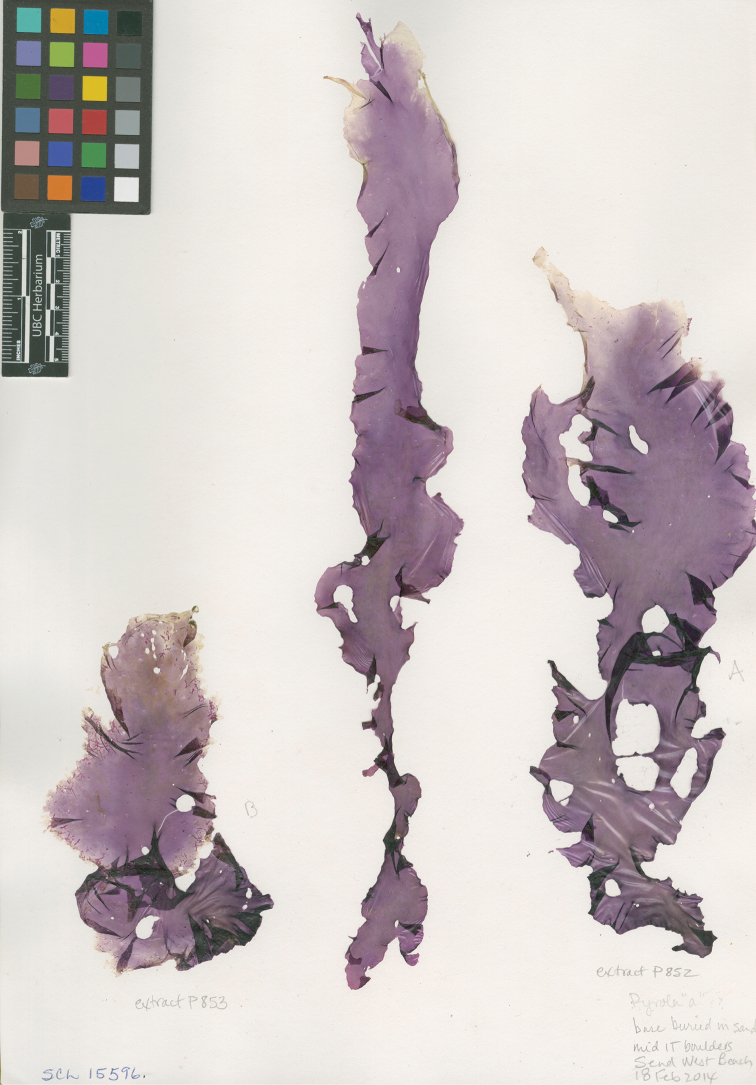
Holotype of *Pyropia columbiensis*. South end of West Beach, Calvert Island, British Columbia, Canada, *S.C. Lindstrom 15596*, 18 Feb 2014, *UBC A90636*.

### Pyropia protolanceolata

Taxon classificationPlantaeBangialesBangiaceae

S.C. Lindstrom & J.R. Hughey sp. nov.

Fig. 4

#### Description.

Thalli linear to lanceolate, base cuneate; 28–65 mm thick; to 1.2 cm wide and 16 cm long; color uniform throughout the thallus except for reproductive areas: dusky rose. Thalli dioecious. Spermatangia in packets 2 × 2 × 8. Zygotosporangial thalli not observed Habitat: very high intertidal, above *Pyropia lanceolata* and *Pyropia montereyensis* when they co-occur. Phenology: Winter to early spring. Distinguished from other species of *Pyropia* by unique *rbc*L and 18S rRNA gene sequences.

#### Holotype.

Saxicolous in the uppermost intertidal, above *Pyropia lanceolata*, northeast side of Morro Rock, Morro Bay, California, USA (35°22.29'N 120°51.98'W), *J.R. Hughey*, 04 Apr 2012, *UBC A90634*. GenBank sequences KP904006 (*rbc*L), KP903909 (SSU).

#### Etymology.

This species is named for its basal position in the phylogeny of the *Pyropia lanceolata* complex.

#### Distribution.

Thus far known only from Spanish Bay, Monterey Peninsula, and northeast side of Morro Rock, Morro Bay, California, USA.

**Figure 4.**
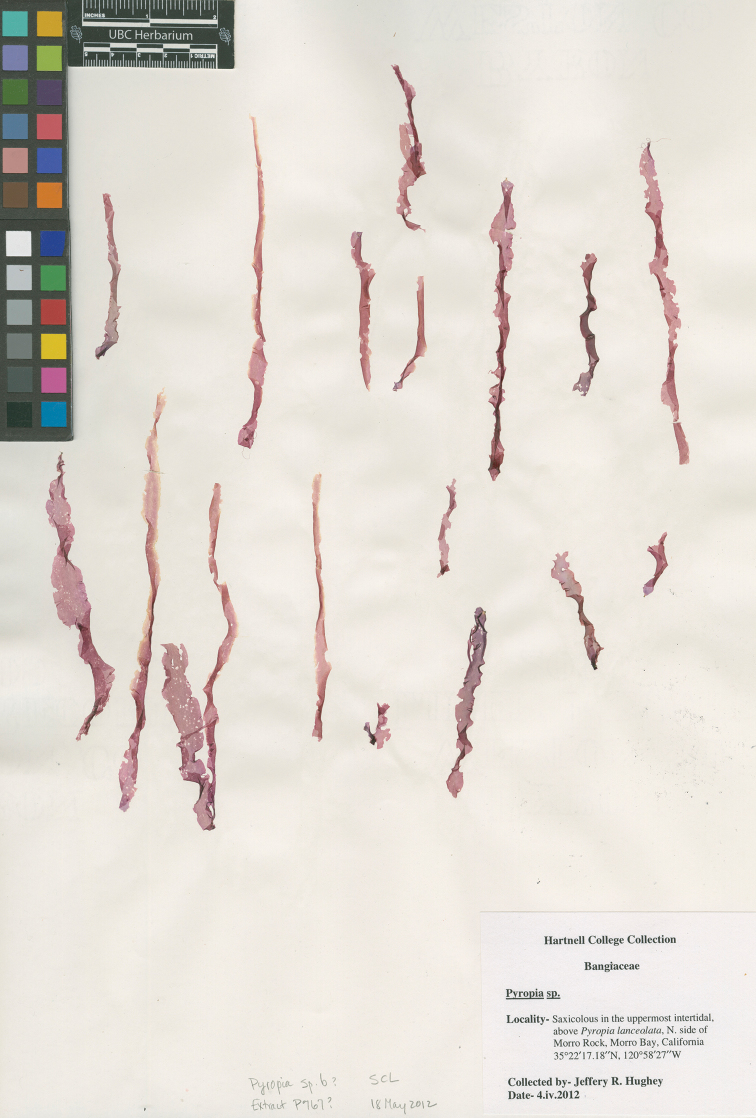
Holotype of *Pyropia protolanceolata*. Northeast side of Morro Rock, Morro Bay, California, USA, *J.R. Hughey*, 04 Apr 2012, *UBC A90634*.

### Pyropia bajacaliforniensis

Taxon classificationPlantaeBangialesBangiaceae

L.E. Aguilar Rosas & J.R. Hughey sp. nov.

Fig. 5

#### Description.

Thalli broadly lanceolate to ovate, sometimes irregularly lobed, base becoming cordate with age; 45–115 mm thick; 1–5 cm wide to at least 15 cm long; monostromatic, with one or two chloroplasts per cell; margin ruffled, often irregular in outline; color pale dusky pink (in California) or lilac gray (Baja California). Monoecious. Spermatangial packets 4 × 4 × 8, cream-colored, variable in shape, mostly marginal in distal portion of thalli but sometimes forming submarginal streaks. Zygotosporangial packets 2–4 × 2–4 × 2–4, appearing as small pinkish speckles because of intermixing of reproductive and vegetative cells. Habitat: upper intertidal rock. Phenology: late winter to late spring. Distinguished from other species of *Pyropia* by unique *rbc*L and 18S rRNA gene sequences.

#### Holotype.

Upper intertidal rock, Playa Saldamando, Baja California, Mexico (31°55.60'N 116°45.30'W), *L.E. Aguilar Rosas & R. Aguilar Rosas 764*, 21 May 2002, *UC 1966778*. GenBank sequences HQ687535 (*rbc*L), DQ084424, DQ084425 (SSU).

#### Isotypes.

*UC 1966778*, *UBC A90700*.

#### Etymology.

The specific epithet refers to the provenance of the type material, where it is especially abundant in spring.

#### Distribution.

Moss Landing, California, USA; Playa Saldamando and Faro de San Miguel, Baja California, Mexico.

**Figure 5.**
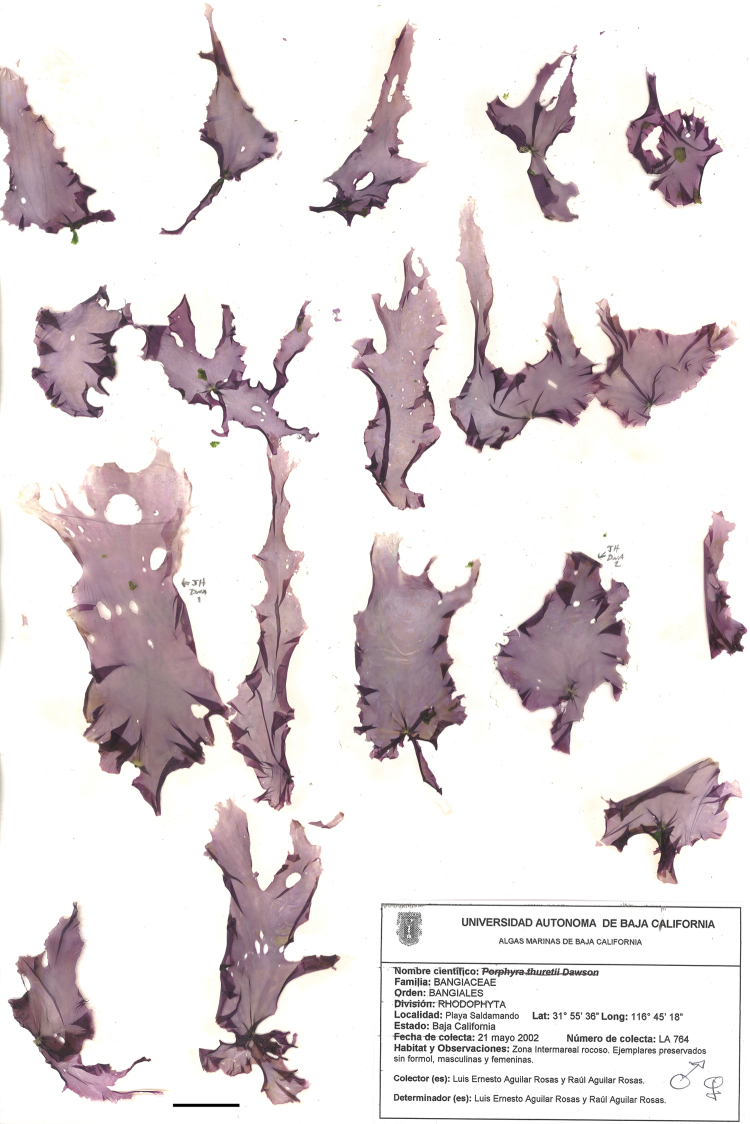
Holotype of *Pyropia bajacaliforniensis*. Playa Saldamando, Baja California, Mexico, *L.E. Aguilar Rosas & R. Aguilar Rosas 764*, 21 May 2002, *UC 1966778*. Scale bar 2.5 cm.

## Discussion

Molecular phylogenetic analysis of the foliose Bangiales indicates that *Pyropia* is the most speciose genus in the order; it also displays the most morphological variation and the widest geographical distribution. Still, there are many geographically restricted clades (Fig. 1, Sutherland et al. 2011). This indicates that much speciation in the order has occurred in particular geographical regions. The *Pyropia lanceolata* clade and its close relatives (*Pyropia kanakaensis*, the *Pyropia nereocystis* clade) are an example of a geographically restricted clade, with species known thus far only from the northeast Pacific, from Baja California, Mexico, to the Aleutian Islands, Alaska. Several of the species are highly restricted geographically: *Pyropia bajacaliforniensis* (in the related *Pyropia nereocystis* clade) is known only from the Moss Landing area of Monterey Bay, CA, and northern Pacific Baja California. Other species are limited to particular areas of coastal California: *Pyropia protolanceolata* thus far known only from Morro Bay and Spanish Bay, California, and *Pyropia montereyensis* from southern to northern California south of Cape Mendocino. In contrast, *Pyropia lanceolata* and especially *Pyropia pseudolanceolata* are widely distributed, occurring from California to Alaska although *Pyropia lanceolata* is replaced by *Pyropia pseudolanceolata* at many sites from British Columbia north. As with all geographic records, these are based on collections to date and are subject to revision due to both more intense collecting efforts in the region as well as changes in distributions due to changing environmental conditions.

The phylogeny of this group of related species suggests a number of patterns that have occurred in the evolution of some of the species. For example, the diplastidial condition in vegetative cells of *Pyropia kanakaensis* has also been observed in species in the *Pyropia lanceolata* complex (Smith and Hollenberg 1943, Lindstrom and Cole 1992b), where division of the chloroplast seems to precede by days or even weeks cell division associated with reproductive cell formation. In species of the *Pyropia lanceolata* clade, the two chloroplasts remain close together (Smith and Hollenberg 1943, Fig. 10) whereas they move to opposite ends of the cell in *Pyropia kanakaensis* (Mumford 1973).

Although the habitat of *Pyropia nereocystis* as an obligate epiphyte on the kelp *Nereocystis* is unique, and *Pyropia kanakaensis* occurs primarily in the lower mid intertidal, the remaining species have adapted to the rigors of the mid to high intertidal. In the *Pyropia lanceolata* clade proper, *Pyropia lanceolata*, *Pyropia pseudolanceolata*, and *Pyropia protolanceolata* are mostly restricted to the high intertidal and are among the highest-occurring species of seaweeds, as are *Pyropia bajacaliforniensis* and *Pyropia* sp. in the *Pyropia nereocystis* clade. *Pyropia fallax* can occur in the high intertidal but also extends into the mid intertidal, where its sister taxon, *Pyropia conwayae*, is found. Where they co-occur, *Pyropia conwayae* usually occurs at a slightly lower elevation than *Pyropia lanceolata*. *Pyropia columbiensis* and *Pyropia montereyensis* also occur primarily in the upper mid to high intertidal although perhaps not as high as *Pyropia lanceolata* and others. Exact elevation of occurrence depends on many factors such as wave exposure, direction the rock is facing as well as season and latitude (and longitude for northern populations). Although thalli can be common on bedrock, when that is the predominant habitat in an area, all of the species can also be abundant on rock protruding from wave-swept sandy shores.

Whereas *Pyropia bajacaliforniensis* and *Pyropia kanakaensis* are spring and spring-summer species, respectively (appearing on the shore ~April, disappearing in June in the case of the former, and persisting as late as November for the latter), the remaining species, including *Pyropia nereocystis*, appear to be winter-spring species, reaching their peak abundance from February to April, and then depending on the species and the location, disappearing from the shore from April to August or later (these later dates occurring for populations near the northern limits of the species).

Because of their similar morphologies, habitats, seasonalities and overlapping distributions, species in this complex have been frequently confused. Much of what has been published on *Pyropia pseudolanceolata* in particular has actually applied to different species. For example, the haploid chromosome number reported by Mumford and Cole (1977) for this species was actually for *Porphyra mumfordii*, and the culture conditions for conchocelis growth and maturation reported by Waaland et al. (1990) were probably for *Pyropia conwayae*. Moreover, the *rbc*L sequence reported for this species by Lindstrom and Fredericq (2003) was that of *Pyropia lanceolata*, as were the culture conditions reported for conchospore release (Lindstrom et al. 2008).

There have also been problems with the identity of *Pyropia lanceolata*. Krishnamurthy (1972) lectotypified Porphyra perforata f. lanceolata, the basionym of *Porphyra lanceolata*, with UC 95720 (collected by Setchell in Carmel Bay, California on 11 Jan 1899), but Lindstrom and Cole (1992b) felt that the specimens on the sheet did not accord with Smith’s description or with the major portion of Setchell & Hus’ description. They therefore designated MO 24356 in UC (Lindstrom and Cole 1992b, Fig. 8), collected by H.T.A. Hus at Land’s End, San Francisco, California, as lectotype since that collection better fit with the original description. The latter contains two outer specimens that are linear in habit, and four inner specimens that are lanceolate. Since modern DNA methods allow the sequencing of historic material, we sequenced a 251 bp region of the *rbc*L gene for the two outer and two inner specimens on MO 24356, a single specimen on UC 95720 (https://ucjeps.cspace.berkeley.edu/ucjeps_project/imageserver/blobs/c68a16ad-5ba5-4c15-8d8d/derivatives/OriginalJpeg/content), which all showed a similar morphology, as well as five of the six specimens on the type sheet of *Pyropia hiberna* (UBC A80269: http://bridge.botany.ubc.ca/herbarium/details.php?db=ubcalgae.fmp12&layout=ubcalgae_web_details&recid=210219&ass_num=A80269), a species closely related if not identical to *Pyropia lanceolata* (Sutherland et al. 2011). Seven of the specimens fell within the variation observed for contemporary collections of *Pyropia lanceolata* (Table 3). Specifically, UC 95720 from Carmel Bay, Monterey Peninsula, and four of the UBC A80269 specimens, all from Pacific Grove, Monterey Peninsula, had sequences identical to the two contemporary specimens from Pacific Grove. The contemporary Monterey Peninsula specimens differed from specimens of *Pyropia lanceolata* from other geographical regions by 0.3%, an amount insufficient to recognize them as a separate species. The distinctness of Monterey Peninsula genotypes within a species has been observed for other organisms (e.g., *Mastocarpus papillatus* (C. Agardh) Kützing, Lindstrom et al. 2011). The two identical inner specimens (one female and one male) on the lectotype sheet of MO 24356 from Land’s End differed by 2 bp from the five Monterey Peninsula specimens noted above, but were identical to other *Pyropia lanceolata* specimens from outside of the Monterey Peninsula. In contrast, the two outer specimens on the same sheet (female far left and male far right) differed from these two inner specimens by 5 bp over the 251 bp region (but by only 3 bp from Monterey Peninsula *Pyropia lanceolata* and by only 2 bp from all *Pyropia conwayae* sequenced). Thus, at this time, we are unable to assign a name to the two outer linear specimens on the sheet of MO 24356. Since MO 24356 is heterotypic, we therefore narrow the lectotypification of MO 24356 to the middle four specimens. Our results also confirm that *Pyropia hiberna* S.C. Lindstrom & K.M. Cole, 1992: 435 is a heterotypic synonym of *Pyropia lanceolata*. The fifth specimen on the type sheet of *Pyropia hiberna* did not match among any described foliose Bangiales sequences but did match a recent collection we recognize here as *Pyropia* sp. (to be described later in a separate paper). *Pyropia lanceolata* was identified as Unknown #3 in Lindstrom (2008).

**Table 3.** Details of sequences of type material (specimen identifier, collection site, collection date, collector, type status, GenBank accession number, and current identification). All sequences represent positions 655–905 in the 1467-bp long *rbc*L gene.

Specimen	Collection site	Collection date	Collector	Type Status	GenBank accession no.	Current identification
UC95720	Carmel Bay, CA	11 Jan 1899	W.A. Setchell	Krishnamurthy (1972) lectotype of *Pyropia lanceolata*	KP904067	*Pyropia lanceolata*
UBC A80269 leftmost	Pacific Grove, CA	29 Dec 1990	S.C. Lindstrom	Holotype of *Pyropia hiberna*	KP904068	*Pyropia lanceolata*
UBC A80269 third from left	Pacific Grove, CA	29 Dec 1990	S.C. Lindstrom	Holotype of *Pyropia hiberna*	KP904069	*Pyropia lanceolata*
UBC A80269 third from right	Pacific Grove, CA	29 Dec 1990	S.C. Lindstrom	Holotype of *Pyropia hiberna*	KP904070	*Pyropia lanceolata*
UBC A80269 second from right	Pacific Grove, CA	29 Dec 1990	S.C. Lindstrom	Holotype of *Pyropia hiberna*	KP904071	*Pyropia lanceolata*
MO24356 in UC center male	Land`s End, San Francisco, CA	08 Feb 1899	H. Hus	Lindstrom and Cole (1992b) lectotype of *Pyropia lanceolata*	KP904072	*Pyropia lanceolata*
MO24356 in UC center female	Land`s End, San Francisco, CA	08 Feb 1899	H. Hus	Lindstrom and Cole (1992b) lectotype of *Pyropia lanceolata*	KP904073	*Pyropia lanceolata*
UBC A80269 second from left	Pacific Grove, CA	29 Dec 1990	S.C. Lindstrom	Holotype of *Pyropia hiberna*	KP904074	*Pyropia* sp.
MO24356 in UC left male	Land`s End, San Francisco, CA	08 Feb 1899	H. Hus	Lindstrom and Cole (1992b) lectotype of *Pyropia lanceolata*	KP904075	probably *Pyropia conwayae* or *Pyropia lanceolata*
MO24356 in UC right female	Land`s End, San Francisco, CA	08 Feb 1899	H. Hus	Lindstrom and Cole (1992b) lectotype of *Pyropia lanceolata*	KP904076	probably *Pyropia conwayae* or *Pyropia lanceolata*

In the earlier paper on the *Pyropia lanceolata* complex (Lindstrom and Cole 1992b), they included *Porphyra mumfordii* as one of the species. This was in part because this entity had previously been misidentified as *Pyropia pseudolanceolata* (Conway et al. 1975, Mumford and Cole 1977). Subsequent DNA sequencing studies have shown that these species are unrelated (Lindstrom and Fredericq 2003, Lindstrom 2008), despite the fact that *Pyropia mumfordii* continues to be easily confused with species in the *Pyropia lanceolata* complex in the field because of similar habitat, seasonality and habit (see Lindstrom and Cole 1992b for a detailed comparison of these species). In conjunction with the present study, we have extended the range of *Pyropia mumfordii* south to Pescadero State Park, California, and north to Calvert Island, British Columbia (Suppl. material 1).

As noted above, the species in the *Pyropia lanceolata* clade show little morphological differentiation. Therefore, the following key to species in this clade relies heavily on geographic distribution and on modest differences in seasonality and elevation on the shore.

**Table:** 

1	Blade oblong or lanceolate, monoecious, sexes intermixed on thalli	***Pyropia fallax***
–	Blade ovate or lanceolate, usually dioecious, if monoecious, sectored	**2**
2	Mid to upper mid intertidal, often associated with sand, in late winter and spring	**3**
–	High intertidal to supralittoral, usually on bedrock, winter to very early spring	**5**
3	Mid intertidal, from Land’s End, San Francisco, California, to Tofino, British Columbia, but most common on the Oregon coast and along the Strait of Juan de Fuca	***Pyropia conwayae***
–	Mid to upper mid intertidal, common on exposed coastlines	**4**
4	Known from Cape Mendocino north in California and on Calvert Island, central coast of British Columbia	***Pyropia columbiensis***
–	Known from just south of Ventura Beach north to the Monterey Peninsula and from Fort Bragg, California	***Pyropia montereyensis***
5	Known only from Spanish Bay, Monterey Peninsula, and northeast of Morro Rock, Morro Bay, California	***Pyropia protolanceolata***
–	Widely distributed from California to Alaska	**6**
6.	Common high intertidal winter species in California (isolated populations at Whiffen Spit and Calvert I., BC, and Sitka Sound, AK)	***Pyropia lanceolata***
–	Common high intertidal winter species from Oregon to Alaska	***Pyropia pseudolanceolata***

## Supplementary Material

XML Treatment for Pyropia montereyensis

XML Treatment for Pyropia columbiensis

XML Treatment for Pyropia protolanceolata

XML Treatment for Pyropia bajacaliforniensis

## Supplementary materials

Supplementary material 1 Data for additional sequenced specimens Data type: List Explanation note: List of specimens by species, including collection data and GenBank accession numbers. This dataset is made available under the Open Database License (http://opendatacommons.org/licenses/odbl/1.0/). The Open Database License (ODbL) is a license agreement intended to allow users to freely share, modify, and use this Dataset while maintaining this same freedom for others, provided that the original source and author(s) are credited.Sandra C. Lindstrom, Jeffery R. Hughey, Luis E. Aguilar Rosas

## References

[B1] Agardh JG (1883) Till algernes systematik. Nya bidrag. (Tredje afdelningen.). Lunds Universitets Års-Skrift, Afdelningen for Mathematik och Naturvetenskap 9(2): 1–177 + 4 plates.

[B2] Blankinship JW Keeler CA (1892) On the natural history of the Farallon Islands. Zoe 3: 144–165.

[B3] Buc A Diallo AB Makarenkov V (2012) T-REX: a web server for inferring, validating and visualizing phylogenetic trees and networks. Nucleic Acid Research 40(W1): W573–W579. doi: 10.1093/nar/gks485 10.1093/nar/gks485 PMC3394261 22675075

[B4] Conway E Mumford TF Jr Scagel RF (1975) The genus *Porphyra* in British Columbia and Washington. Syesis 8: 185–244.

[B5] Croom M Wolotira R Henwood W (1995) Marine Region 15: Northeast Pacific. A global representative system of marine protected areas. Vol. 4 Great Barrier Reef Marine Park Authority, The World Bank, The World Conservation Union (IUCN), Washington, D.C., 55–107.

[B6] Field D Tiwari B Booth T Houten S Swan D Bertrand N Thurston M (2006) Open software for biologists: from famine to feast. Nature Biotechnology 24: 801–803. doi: 10.1038/nbt0706-801 10.1038/nbt0706-801 16841067

[B7] Garbary DJ Hansen GI Scagel RF (1980) The marine algae of British Columbia and northern Washington: Division Rhodophyta (red algae), Class Bangiophyceae. Syesis 13: 137–195.

[B8] Hall TA (1999) BioEdit: a user-friendly biological sequence alignment editor and analysis program for Windows 95/98/NT. Nucleic Acids Symposium Series 41: 95–98.

[B9] Huelsenbeck JP Ronquist F Nielsen R Bollback JP (2001) Bayesian inference of phylogeny and its impact on evolutionary biology. Science 294: 2310–2314. doi: 10.1126/science.1065889 11743192 10.1126/science.1065889

[B10] Hughey JR Gabrielson PG (2012) Comment on “Acquiring DNA sequence data from dried archival red algae (Florideophyceae) for the purpose of applying available names to contemporary genetic species: a critical assessment”. Botany 90: 191–203. doi: 10.1139/b2012-102

[B11] Hus HTA (1900) Preliminary notes on west-coast *Porphyra* s. Zoe 5: 61–70.

[B12] Hus HTA (1902) An account of the species of *Porphyra* found on the Pacific coast of North America. Proceedings of the California Academy of Sciences, Ser. 3, Botany 2: 173–241.

[B13] Krishnamurthy V (1972) A revision of the species of the algal genus *Porphyra* occurring on the Pacific coast of North America. Pacific Science 26: 24–49.

[B14] Kucera H Saunders GW (2012) A survey of Bangiales (Rhodophyta) based on multiple molecular markers reveals cryptic diversity. Journal of Phycology 48: 869–882. doi: 10.1111/j.1529-8817.2012.01193.x 10.1111/j.1529-8817.2012.01193.x 27008998

[B15] Le Gall L Saunders GW (2010) DNA barcoding is a powerful tool to uncover algal diversity: a case study of the Phyllophoraceae (Gigartinales, Rhodophyta) in the Canadian flora. Journal of Phycology 46: 374–389. doi: 10.1111/j.1529-8817.2010.00807.x

[B16] Lindstrom SC (1993) Intraspecific and interspecific genetic variation in species of *Porphyra* (Rhodophyta, Bangiales) from British Columbia and adjacent waters. Journal of Applied Phycology 5: 53–62. doi: 10.1007/BF02182422

[B17] Lindstrom SC (2008) Cryptic diversity, biogeography and genetic variation in Northeast Pacific species of *Porphyra* sensu lato (Bangiales, Rhodophyta). Journal of Applied Phycology 20: 951–962. doi: 10.1007/s10811-008-9313-9

[B18] Lindstrom SC Cole KM (1990) *Porphyra fallax*, a new species of Rhodophyta from British Columbia and northern Washington. Japanese Journal of Phycology 38(4): 371–376.

[B19] Lindstrom SC Cole KM (1992a) Relationships between some North Atlantic and North Pacific species of *Porphyra* (Bangiales, Rhodophyta): evidence from isozymes, morphology, and chromosomes. Canadian Journal of Botany 70(7): 1355–1363. doi: 10.1139/b92-170

[B20] Lindstrom SC Cole KM (1992b) The *Porphyra lanceolata*-*P. pseudolanceolata* (Bangiales, Rhodophyta) complex unmasked: recognition of new species based on isozymes, morphology, chromosomes and distributions. Phycologia 31(5): 431–448. doi: 10.2216/i0031-8884-31-5-431.1

[B21] Lindstrom SC Cole KM (1992c) A revision of the species of *Porphyra* (Rhodophyta: Bangiales) occurring in British Columbia and adjacent waters. Canadian Journal of Botany 70(10): 2066–2075. doi: 10.1139/b92-256

[B22] Lindstrom SC Fredericq S (2003) *rbc*L gene sequences reveal relationships among north-east Pacific species of *Porphyra* (Bangiales, Rhodophyta) and a new species, *P. aestivalis*. Phycological Research 51(3): 211–224. doi: 10.1111/j.1440-1835.2003.tb00189.x

[B23] Lindstrom SC Conitz JM Hall S Stekoll MS (2008) Induction of conchospore release: ecotypic variation in northeast Pacific species of *Porphyra*. Journal of Applied Phycology 20: 331–340. doi: 10.1007/s10811-007-9261-9

[B24] Lindstrom SC Hughey JR Martone PT (2011) New, resurrected and redefined species of *Mastocarpus* (Phyllophoraceae, Rhodophyta) from the northeast Pacific. Phycologia 50(6): 661–683. doi: 10.2216/10-38.1

[B25] Mols-Mortensen A Neefus CD Nielsen R Gunnarsson K Egilsdóttir S Pedersen PM Brodie J (2012) New insights into the biodiversity and genetic relationships of foliose Bangiales (Rhodophyta) in Iceland and the Faroe Islands. European Journal of Phycology 47(2): 146–159. doi: 10.1080/09670262.2012.666678

[B26] Mumford TF Jr (1973) A new species of *Porphyra* from the west coast of North America. Syesis 6: 239–242.

[B27] Mumford TF Jr Cole KM (1977) Chromosome numbers for fifteen species in the genus *Porphyra* (Bangiales, Rhodophyta) from the west coast of North America. Phycologia 16(4): 373–377. doi: 10.2216/i0031-8884-16-4-373.1

[B28] Nelson WA Broom JES (2010) The identity of *Porphyra columbina* (Bangiales, Rhodophyta) originally described from the New Zealand subantarctic islands. Australian Systematic Botany 23: 16–26. doi: 10.1071/SB09032

[B29] Oliveira MC Kurniawan J Bird CJ Rice EL Murphy CA Singh RK Gutell RR Ragan MA (1995) A preliminary investigation of the order Bangiales (Bangiophycidae, Rhodophyta) based on sequences of nuclear small-subunit ribosomal RNA genes. Phycological Research 43(2): 71–79. doi: 10.1111/j.1440-1835.1995.tb00007.x

[B30] Ronquist F Huelsenbeck JP (2003) MRBAYES 3: Bayesian phylogenetic inference under mixed models. Bioinformatics 19: 1572–1574. doi: 10.1093/bioinformatics/btg180 12912839 10.1093/bioinformatics/btg180

[B31] Smith GM Hollenberg GJ (1943) On some Rhodophyceae from the Monterey Peninsula, California. American Journal of Botany 30: 211–222. doi: 10.2307/2437242

[B32] Stamatakis A (2006) RAxML-VI-HPC: maximum-likelihood based phylogenetic analyses with thousands of taxa and mixed models. Bioinformatics 22: 2688–2690. doi: 10.1093/bioinformatics/btl446 16928733 10.1093/bioinformatics/btl446

[B33] Sutherland JE Lindstrom SC Nelson WA Brodie J Lynch MDJ Hwang MS Choi H-G Miyata M Kikuchi N Oliveira MC Farr T Neefus C Mols-Mortensen A Milstein D Müller KM (2011) A new look at an ancient order: generic revision of the Bangiales (Rhodophyta). Journal of Phycology 47: 1131–1151. doi: 10.1111/j.1529-8817.2011.01052.x 10.1111/j.1529-8817.2011.01052.x 27020195

[B34] Swofford D (2002) PAUP*: Phylogenetic Analysis Using Parsimony (*and Other Methods), Version 4.0b20. Sinauer Associates, Sunderland, MA.

[B35] Valentine JW (1966) Numerical analysis of marine molluscan ranges on the extratropical northeastern Pacific shelf. Limnology and Oceanography 11: 198–211. doi: 10.4319/lo.1966.11.2.0198

[B36] Waaland JR Dickson LG Duffield ECS (1990) Conchospore production and seasonal occurrence of some *Porphyra* species (Bangiales, Rhodophyta) in Washington State. Hydrobiologia 204/205: 453–459. doi: 10.1007/BF00040270

