# Supplementary material for: Four new species of Pyropia (Bangiales, Rhodophyta) from the west coast of North America: the Pyropia lanceolata species complex updated
Source: PhytoKeys. 2015 Jun 25;(52):1–22. doi: 10.3897/phytokeys.52.5009 (PMC4549882; doi:10.3897/phytokeys.52.5009)
Supplement: Supplementary material 1 — Data for additional sequenced specimens [file phytokeys-052-001-s001.pdf]

Supplementary table. Additional specimens for which the *rbc*L gene was sequenced for this study. (18S rRNA gene sequences are noted in parentheses as SSU.) These are identified by extract no., collection location, collection date, collector, collector number, and GenBank number(s). Identity with specimen sequences listed in Table 1 is indicated. All herbarium vouchers are deposited in *UBC* unless noted otherwise.

Pyropia fallax

Same as P172:

SOG, Wreck Beach, Vancouver, BC, 17 Dec 1992, S.C. Lindstrom, SCL 10285, AF452429
P729, Ebey's Landing, WA, 06 May 2011, S.C. Lindstrom, SCL 14663, KP903929
P730, Ebey's Landing, WA, 06 May 2011, S.C. Lindstrom, SCL 14664, KP903930
P731, Ebey's Landing, WA, 06 May 2011, S.C. Lindstrom, SCL 14665, KP903931

Same as P544:

P371, Volcano Bay, Unalaska I., AK, 09 Jun 2005, S.C. Lindstrom, SCL 12286, EU223071

Same as P557:

P204, Akun I., AK, 31 Jul 2005, S.C. Lindstrom, SCL 12637, EU223058
P206, Akun I., AK, 31 Jul 2005, S.C. Lindstrom, SCL 12634, EU223059
P207, Akun I., AK, 31 Jul 2005, S.C. Lindstrom, SCL 12641, EU223060
P208, Akun I., AK, 31 Jul 2005, S.C. Lindstrom, SCL 12640, EU223061
P271, Outside Beach, AK, 06 Jun 1996, Mandy Lindeberg collection (in ALAJ), EU223065
P274, Gull I., Kachemak Bay, AK, 06 Jun 1996, S.C. Lindstrom, SCL 8867, EU223066
P356, Lucy I., BC, 18 Apr 2007, S.C. Lindstrom, SCL 13134, EU223067
P357, Lucy I., BC, 18 Apr 2007, S.C. Lindstrom, SCL 13135, EU223068
P358, Lucy I., BC, 18 Apr 2007, S.C. Lindstrom, SCL 13134, KP903912
P359, Lucy I., BC, 18 Apr 2007, S.C. Lindstrom, SCL 13260, EU223069
P368, Humpback Bay, Unalaska I., AK, 06 Jun 2005, S.C. Lindstrom, SCL 12210, EU223070
P372, Akun I., AK, 31 Jul 2005, S.C. Lindstrom, SCL 12639, EU223072
P379, Akutan Harbor, 30 Jul 2004, S.C. Lindstrom, SCL 11503, KP903913
P466, Atka I., AK, 15 Jul 2007, M.R. Lindeberg, ALEUT07_356 (silica gel only), KP903915
P469, Chugach Bay, AK, 09 Jul 2002, S.C. Lindstrom, SCL 10220, KP903916
P527, Chichagof Harbor, Attu I., AK, 04 Jun 2008, S.C. Lindstrom, SCL 13508, KP903918
P682, NW Barkley Sd, BC, 16 May 2010, S.C. Lindstrom, no herbarium voucher, KP903925
P702, Kiukpalik I., AK, 30 Jul 1993, G.I. Hansen, no herbarium voucher, KP903926
P703, Sunset Beach, OR, 06 Apr 2008, S.C. Lindstrom, no herbarium voucher, KP903927
P704, Harris Beach State Park, OR, 03 Apr 1994, S.C. Lindstrom, SCL 9066?, KP903928
PW2, Driftwood Bay, Unalaska I., AK, 08 Jul 2006, M.R. Lindeberg, ALEUT06_134, EU223080
PW9, Skan Bay, Unalaska I., AK, 07 Jun 2005, S.C. Lindstrom, SCL 12229, EU223081
PW24, Pumicestone Bay, Unalaska I., AK, 03 Jun 2005, S.C. Lindstrom, SCL 12166, EU223083
P789, North Beach, Calvert I., BC, 04 Jul 2012, S.C. Lindstrom, SCL 14994C, KP903933

Same as P577:

P219, Hallo Bay, AK, 17 Jun 2003, S.C. Lindstrom, SCL 10629, EU223062
P221, Katmai Bay, AK, 12 Jun 2003, S.C. Lindstrom, SCL 10326, EU223063
P436, Pasagshak Bay, Kodiak I., AK, 19 May 2005, S.C. Lindstrom, SCL 13261, EU223079
P444, Kiukpalik I., AK, 16 Jun 2003, S.C. Lindstrom, SCL 10763, KP903914

P546, Avatanak I., AK, 12 Jun 2008, S.C. Lindstrom, SCL 13732, KP903921, KP903905 (SSU)
P578, Takli I., AK, 06 Jul 2009, M.R. Lindeberg, [UBC A89045], KP903924
P840, Japonski I., AK, 25 Jun 2013, S.C. Lindstrom, SCL 15447, KP903947
PW10, Cannery Bay, Unalaska I., AK, 05 Jun 2005, S.C. Lindstrom, SCL 12197, EU223082
PW28, Shakun I., AK, 16 Jun 2003, S.C. Lindstrom, SCL 10806, EU223084
Same as P815:
P817, Fourth Beach, Calvert I., BC, 26 May 2013, S.C. Lindstrom, SCL 15303, KP903937
Same as P820:
P807, West Beach boulder, Calvert I., BC, 21 May 2013, S.C. Lindstrom, SCL 15263, KP903934
Same as 851:
P789, North Beach, Calvert I., BC, 04 Jul 2012, S.C. Lindstrom, SCL 14994C, KP903933
P808, West Beach boulder, Calvert I., BC, 21 May 2013, S.C. Lindstrom, SCL 15264, KP903935
P818, Fourth Beach, Calvert I., BC, 26 May 2013, S.C. Lindstrom, SCL 15302, KP903938
P819, Fourth Beach, Calvert I., BC, 26 May 2013, S.C. Lindstrom, SCL 15301, KP903939
P821, Fourth Beach, Calvert I., BC, 26 May 2013, S.C. Lindstrom, SCL 15303, KP903941
P823, Fourth Beach, Calvert I., BC, 26 May 2013, S.C. Lindstrom, SCL 15300, KP903942
P824, Fourth Beach, Calvert I., BC, 26 May 2013, S.C. Lindstrom, SCL 15300, KP903943
P826, Fifth Beach, Calvert I., BC, 26 May 2013, S.C. Lindstrom, SCL 15296, KP903945
P832, Japonski I., Sitka Sound, AK, 23 Jun 2013, S.C. Lindstrom, SCL 15401, KP903946
P864, West Beach, Calvert I., BC, 18 Feb 2014, S.C. Lindstrom, SCL 15603, KP903949
P865, West Beach, Calvert I., BC, 18 Feb 2014, S.C. Lindstrom, SCL 15604, KP903950
P897, Fourth Beach, Calvert I., 20 Feb 2014, S.C. Lindstrom, SCL 15639, KP903951
P898, Fourth Beach, Calvert I., 20 Feb 2014, S.C. Lindstrom, SCL 15640, KP903952
P899, Fifth Beach, Calvert I., BC, 20 Feb 2014, S.C. Lindstrom, SCL 15641, KP903953
P900, Fifth Beach, Calvert I., BC, 20 Feb 2014, S.C. Lindstrom, SCL 15641, KP903954
P902, Fourth Beach, Calvert I., BC, 20 Feb 2014, S.C. Lindstrom, SCL 15643, KP903955
P922, North Beach, Calvert I., BC, 13 Jun 2014, S.C. Lindstrom, SCL 15675, KP903956

Pyropia conwayae
Same as P589:
P131, Baker Beach, CA, 25 May 2002, S.C. Lindstrom, SCL 11404, EU223040
P334, Baker Beach, CA, 25 May 2002, S.C. Lindstrom, SCL 11404, KP903963
P389, Tonquin Park, Tofino, BC, 17 May 2007, S.C. Lindstrom, SCL 13190, EU223041
P390, Chesterman Beach, BC, 17 May 2007, S.C. Lindstrom, SCL 13208, EU223042
P391, Chesterman Beach, BC, 17 May 2007, S.C. Lindstrom, SCL 13208, EU223043
P431, French Beach, BC, 12 Mar 2007, S.C. Lindstrom, SCL 13109, EU223045
P441, Chesterman Beach, BC, 17 May 2007, S.C. Lindstrom, SCL 13204, EU223046
P445, Chesterman Beach, BC, 17 May 2007, S.C. Lindstrom, SCL 13206, EU223047
P486, Mussel Rock, Cape Mendocino, CA, 08 Apr 2008, SCL 13356, KP903959
P493, Trinidad State Beach, CA, 12 Apr 2008, F.J. Shaughnessy, HSC, KP903958
P583, Trinidad Head, CA, 01 Jan 2010, S. Augyte, HSC, KP903960

Pyropia montereyensis
Same as P603:
P607, Fort Bragg, CA, 15 Feb 2010, S.C. Lindstrom, SCL 14315, KP903965
P608, Fort Bragg, CA, 15 Feb 2010, S.C. Lindstrom, SCL 14316, KP903966
P759, Spanish Bay, CA, 02 Feb 2012, J.R. Hughey, UBC A90632, KP903969
P760, Carmel River St. Beach, CA, 02 Feb 2012, J.R. Hughey, UBC A90628, A90629, A90630, KP903970, KP903907 (SSU)
P763, Spanish Bay, Monterey Pen., CA, 02 Feb 2012, J.R. Hughey, UBC A90632, KP903972
P768, Carmel River St. Beach, CA, 02 Feb 2012, J.R. Hughey, UBC A90628, A90629, A90630, KP903974
UC1966780, Carmel River St. Beach, CA, 15 Jan 2012, J.R. Hughey, UC 1966780, KP903978
Same as P645:
P761, Morro Rock, CA, 04 Apr 2012, J.R. Hughey, UBC A90631, KP903971
P765, Morro Rock, CA, 04 Apr 2012, J.R. Hughey, UBC A90631, KP903973
JN028999, Jade Cove, CA, 18 May 2010, B. Clarkston & K. Hind, GWS021820 in UNB
Same as P763:
P769, Spanish Bay, Monterey Pen., CA, 02 Feb 2012, J.R. Hughey, UBC A90632, KP903975
P770, Carmel River St. Beach, CA, 02 Feb 2012, J.R. Hughey, UBC A90628, A90629, A90630, KP903976
P771, Spanish Bay, Monterey Pen., CA, 02 Feb 2012, J.R. Hughey, UBC A90632, KP903977

Pyropia columbiensis
Same as P491:
P492, Trinidad State Beach, CA,12 April 2008, F.J. Shaughnessy, Frank #2 in HSC, KP903983
Same as P859:
P485, Cape Mendocino, CA, 08 Apr 2008, S.C. Lindstrom, SCL 13358, KP903981, KP903903 (SSU)
P499, Mussel Rock, Cape Mendocino, CA, 08 Apr 2008, S.C. Lindstrom, SCL 13350, KP903984
P501, Mussel Rock, Cape Mendocino, CA, 08 Apr 2008, S.C. Lindstrom, SCL 13354, KP903985
P502, Mussel Rock, Cape Mendocino, CA, 08 Apr 2008, S.C. Lindstrom, SCL 13352, KP903986
P512, Mussel Rock, Cape Mendocino, CA, 08 Apr 2008, S.C. Lindstrom, no voucher, KP903988
P582, Trinidad Head, CA, 01 Jan 2010, S. Augyte, SA301 in HSC, KP903989, KP903906 (SSU)
P597, Humboldt Bay, north jetty, CA, 15 Apr 2008, S.C. Lindstrom, SCL 14299, KP903990
P739, Fourth Beach, Calvert I., BC, 14 Aug 2011, S.C. Lindstrom, SCL 14746, KP903991
P791, Fifth Beach, Calvert I., BC, 05 Jul 2012, S.C. Lindstrom, SCL 15016, KP903992
P849, West Beach, Calvert I., BC, 18 Feb 2014, S.C. Lindstrom, SCL 15594, KP903993
P850, West Beach, Calvert I., BC, 18 Feb 2014, S.C. Lindstrom, SCL 15594, KP903994
P852, West Beach, Calvert I., BC, 18 Feb 2014, S.C. Lindstrom, SCL 15596, KP903995, KP903910 (SSU)

P853, West Beach, Calvert I., BC, 18 Feb 2014, S.C. Lindstrom, SCL 15596, KP903996
P857, West Beach, Calvert I., BC, 18 Feb 2014, S.C. Lindstrom, SCL 15599, KP903997
P858, West Beach, Calvert I., BC, 18 Feb 2014, S.C. Lindstrom, SCL 15599, KP903998
P859, West Beach, Calvert I., BC, 18 Feb 2014, S.C. Lindstrom, SCL 15599, KP903999
P860, West Beach, Calvert I., BC, 18 Feb 2014, S.C. Lindstrom, SCL 15599, KP904000
P861, West Beach, Calvert I., BC, 18 Feb 2014, S.C. Lindstrom, SCL 15600, KP904001
P862, West Beach, Calvert I., BC, 18 Feb 2014, S.C. Lindstrom, SCL 15601, KP904002
P893, Fifth Beach, Calvert I., BC, 20 Feb 2014, S.C. Lindstrom, SCL 15637, KP904003, KP903911 (SSU)
P901, Fifth Beach, Calvert I., BC, 20 Feb 2014, S.C. Lindstrom, SCL 15642, KP904004

Pyropia lanceolata
Same as P638:
Ppse, John Brown's Beach, AK, 05 Apr 1996, S.C. Lindstrom, ALAJ 828, AF452439
P200, Whiffin Spit, BC, 03 Feb 2002, S.C. Lindstrom, SCL 13271, EU223138
P228, Whiffin Spit, BC, 03 Feb 2002, S.C. Lindstrom, SCL 13271, EU223139
P235, John Brown's Beach, AK, 05 Apr 1996, S.C. Lindstrom, ALAJ 1430, EU223140
P585, Trinidad boat launch ramp, CA, 14 Feb 2010, S.C. Lindstrom, SCL 14277, KP904009
P588, Camel Rock, CA, 14 Feb 2010, S.C. Lindstrom, SCL 14286, KP904010
P590, Humboldt Bay, north jetty, CA, 15 Feb 2010, S.C. Lindstrom, SCL 14290, KP904011
P591, Humboldt Bay, north jetty, CA, 15 Feb 2010, S.C. Lindstrom, 14291, KP904012
P592, Humboldt Bay, north jetty, CA, 15 Feb 2010, S.C. Lindstrom, 14292, KP904013
P593, Humboldt Bay, north jetty, CA, 15 Feb 2010, S.C. Lindstrom, 14293, KP904014
P594, Humboldt Bay, north jetty, CA, 15 Feb 2010, S.C. Lindstrom, 14294, KP904015
P595, Humboldt Bay, north jetty, CA, 15 Feb 2010, S.C. Lindstrom, 14295, KP904016
P596, Humboldt Bay, north jetty, CA, 15 Feb 2010, S.C. Lindstrom, 14296, KP904017
P601, Fort Bragg, CA, 15 Feb 2010, S.C. Lindstrom, SCL 14309, KP904018
P602, Fort Bragg, CA, 15 Feb 2010, S.C. Lindstrom, SCL 14310, KP904019
P606, Fort Bragg, CA, 15 Feb 2010, S.C. Lindstrom, SCL 14314, KP904020
P609, Van Damme State Park, CA, 16 Feb 2010, S.C. Lindstrom, SCL 14318, KP904021
P610, Van Damme State Park, CA, 16 Feb 2010, S.C. Lindstrom, SCL 14319, KP904022
P611, Van Damme State Park, CA, 16 Feb 2010, S.C. Lindstrom, SCL 14320, KP904023
P613, Van Damme State Park, CA, 16 Feb 2010, S.C. Lindstrom, SCL 14322, KP904025
P620, Point Arena, CA, 16 Feb 2010, S.C. Lindstrom, SCL 14330, KP904026
P623, Bodega Marine Laboratory, CA, 16 Feb 2010, S.C. Lindstrom, SCL 14339, KP904027
P624, Bodega Marine Laboratory, CA, 16 Feb 2010, S.C. Lindstrom, SCL 14340, KP904028
P628, Bodega Marine Laboratory, CA, 16 Feb 2010, S.C. Lindstrom, SCL 14346, KP904030
P630, Bodega Marine Laboratory, CA, 16 Feb 2010, S.C. Lindstrom, SCL 14355, KP904031
P631, Fort Point, San Francisco, CA, 17 Feb 2010, S.C. Lindstrom, no voucher, KP904032
P632, Fort Point, San Francisco, CA, 17 Feb 2010, S.C. Lindstrom, no voucher, KP904033
P633, Fort Point, San Francisco, CA, 17 Feb 2010, S.C. Lindstrom, SCL 14359, KP904034
P634, Fort Point, San Francisco, CA, 17 Feb 2010, S.C. Lindstrom, SCL 14360, KP904035
P635, Pescadero State Park, CA, 17 Feb 2010, S.C. Lindstrom, no voucher, KP904036
P636, Pescadero State Park, CA, 17 Feb 2010, S.C. Lindstrom, SCL 14363, KP904037
P647, Kenneth Norris Rancho Marina Reserve, CA, 18 Feb 2010, S.C. Lindstrom, SCL 14380, KP904040

P662, Low Arch, Farallon Is., CA, 07-Feb-2006, K.A. Miller, SEF1-I-04 in UC, KP904041
P663, Raven´s Cliff, Farallon Is., CA, 08-Feb-2006, K.A. Miller, SEF1-II-32 in UC, KP904042
P880, North Beach, Calvert I., BC, 19 Feb 2014, S.C. Lindstrom, SCL 15626, KP904043
P882, North Beach, Calvert I., BC, 19 Feb 2014, S.C. Lindstrom, SCL 15628, KP904044
P894, Fifth Beach, Calvert I., BC, 20 Feb 2014, S.C. Lindstrom, SCL 15638, KP904045
P895, Fifth Beach, Calvert I., BC, 20 Feb 2014, S.C. Lindstrom, SCL 15638, KP904046
P896, Fifth Beach, Calvert I., BC, 20 Feb 2014, S.C. Lindstrom, SCL 15638, KP904047
Same as P641:
P479, Pacific Grove, CA, 31 Dec 2007, P.W. Gabrielson, PWG 1564, KP904007, KP903901 (SSU)

Pyropia pseudolanceolata
Same as P351:
P199, Cape Sitkinak, AK, 24 Jun 2005, S.C. Lindstrom, SCL 12485, EU223150
P205, Surf Bay, Akun I., AK, 31 Jul 2005, S.C. Lindstrom, SCL 12638, KP904048
P348, Lucy I., BC, 18 Apr 2007, S.C. Lindstrom, SCL 13125B, EU223160
P349, Lucy I., BC, 18 Apr 2007, S.C. Lindstrom, SCL 13125B, EU223161
P350, Dundas I., BC, 19 Apr 2007, S.C. Lindstrom, SCL 13136, EU223162
P373, Lucy I., BC, 18 Apr 2007, S.C. Lindstrom, SCL 13125B, EU223164
P543, Surveyor Bay, Unalaska I., AK, 11 Jun 2008, S.C. Lindstrom, SCL 13712, KP904058
Same as P488:
P242V, Marine Gardens, OR, 08 Jan 1993, S.C. Lindstrom, SCL 13275, EU223156
P497, Sunset Beach, OR, 06 Apr 2008, S.C. Lindstrom, SCL 13324, KP904053
Same as P537:
P143, Amalik Bay, AK, 13 Jun 2003, S.C. Lindstrom, SCL 10343, EU223141
P149, Akutan Pt, AK, 30 Jul 2004, S.C. Lindstrom, SCL 11576, EU223142
P179, Rosario Beach, WA, 05 Mar 2005, S.C. Lindstrom, SCL 11576, EU223143
P181, Rosario Beach, WA, 05 Mar 2005, S.C. Lindstrom, SCL 11576, EU223144
P193, Harling Pt, Vancouver I., BC, 05 Feb 2005, S.C. Lindstrom, SCL 12538, EU223145
P195, Izhut Bay, AK, 21 May 2005, S.C. Lindstrom, SCL 11813, EU223146
P196, Big Bay, Shuyak I., AK, 24 May 2005, S.C. Lindstrom, SCL 11970, EU223147
P197, Sedanka Pt, Unalaska I., AK, 03 Jun 2005, S.C. Lindstrom, SCL 12136, EU223148
P198, Humpback Bay, Unalaska I., AK, 06 Jun 2005, S.C. Lindstrom, SCL 12208, EU223149
P227, Skan Bay, Unalaska I., AK, 07 Jun 2005, S.C. Lindstrom, SCL 12234, EU223151
P231, Deadman Bay, WA, 23 Jan 2002, S.C. Lindstrom, SCL 13273, EU223152
P233, French Beach, BC, 20 Apr 1999, S.C. Lindstrom, SCL 13274, EU223153
P239, Botany Bay, BC, 03 Mar 2006, S.C. Lindstrom, SCL 12864, EU223154
P242, Fishboat Bay, BC, 06 Jan 2006, S.C. Lindstrom, SCL 12860, EU223155
P253, Perevalnie Passage, AK, 23 May 2005, S.C. Lindstrom, SCL 11885, EU223157
P336, Whiffin Spit, BC, 12 Mar 2007, S.C. Lindstrom, SCL 13111, EU223158
P337, Whiffin Spit, BC, 12 Mar 2007, S.C. Lindstrom, SCL 13111, EU223158
P338, Whiffin Spit, BC, 12 Mar 2007, S.C. Lindstrom, SCL 13111, EU223158
P339, Whiffin Spit, BC, 12 Mar 2007, S.C. Lindstrom, SCL 13111, EU223158
P346, Harling Point, BC, 12 Mar 2007, S.C. Lindstrom, SCL 13115, EU223159
P418, Big Bay, Shuyak I., AK, 24 May 2005, S.C. Lindstrom, SCL 11973, EU223166

P421, Perevalnie Passage, AK, 23 May 2005, S.C. Lindstrom, SCL 11881, EU223167
P465, Kanaga Sd, Kanaga I., AK, 08 Jul 2007, M.R. Lindeberg, ALEUT07_305, KP904051
P500, Battery Point Light, Crescent City, CA, 06 Apr 2008, S.C. Lindstrom, SCL 13329, KP904055, KP903904 (SSU)
P537, Alaid I., AK, 07 Jun 2008, S.C. Lindstrom, SCL 13630, KP904056
P538, Alaid I., AK, 07 Jun 2008, S.C. Lindstrom, SCL 13630, KP904057
P581, Deadman Bay, WA, 11 Dec 2009, S.C. Lindstrom, SCL 14248, KP904059
P670, Avatanak I., AK, 12 Jun 2008, S.C. Lindstrom, SCL 13729, KP904060

Pyropia kanakaensis
Same as P132:
P105, Baker Beach, CA, 25 May 2002, S.C. Lindstrom, SCL 11405, EU223097
P322, Baker Beach, CA, 25 May 2002, S.C. Lindstrom, SCL 11412, EU223100

Pyropia nereocystis
Same as P814, P827:
P262, Umnak I., AK, 18 Jul 2006, Mandy Lindeberg, #333, EU223115
P325, Ushugat I., AK, 25Aug 2006, S.C. Lindstrom, SCL 13101, 13102, EU223117
P721, Sunshine Cove, Juneau, AK, 19 Apr 2011, S.C. Lindstrom, SCL 14613, KP904061
P877, Calvert I., BC, 19 Feb 2014, S.C. Lindstrom, SCL 15622, KP904064

Porphyra mumfordii
P506, Sunset Beach, OR, 06 Apr 2008, S.C. Lindstrom, SCL 13313, KP904077
P587, Camel Rock, CA, 14 Feb 2010, S.C. Lindstrom, SCL 14285, KP904078
P598, Humboldt Bay, north jetty, CA, 15 Feb 2010, S.C. Lindstrom, SCL 14300, KP904079
P599, Humboldt Bay, north jetty, CA, 15 Feb 2010, S.C. Lindstrom, SCL 14301, KP904080
P600, Humboldt Bay, north jetty, CA, 15 Feb 2010, S.C. Lindstrom, SCL 14305, KP904081
P614, Point Arena, CA, 16 Feb 2010, S.C. Lindstrom, SCL 14324, KP904082
P615, Point Arena, CA, 16 Feb 2010, S.C. Lindstrom, SCL 14325, KP904083
P616, Point Arena, CA, 16 Feb 2010, S.C. Lindstrom, SCL 14326, KP904084
P617, Point Arena, CA, 16 Feb 2010, S.C. Lindstrom, SCL 14327, KP904085
P618, Point Arena, CA, 16 Feb 2010, S.C. Lindstrom, SCL 14328, KP904086
P621, Point Arena, CA, 16 Feb 2010, S.C. Lindstrom, SCL 14331, KP904087
P622, Bodega Marine Lab, CA, 16 Feb 2010, S.C. Lindstrom, SCL 14338, KP904088
P637, Pescadero State Park, CA, 17 Feb 2010, S.C. Lindstrom, SCL 14364, KP904089
P854, Calvert I., BC, 18 Feb 2014, S.C. Lindstrom, SCL 15597, KP904090
P855, Calvert I., BC, 18 Feb 2014, S.C. Lindstrom, SCL 15597, KP904091
P863, Calvert I., BC, 18 Feb 2014, S.C. Lindstrom, SCL 15602, KP904092
P871, Calvert I., BC, 19 Feb 2014, S.C. Lindstrom, SCL 15618, KP904093
P872, Calvert I., BC, 19 Feb 2014, S.C. Lindstrom, SCL 15618, KP904094
P873, Calvert I., BC, 19 Feb 2014, S.C. Lindstrom, SCL 15618, KP904095
P881, Calvert I., BC, 19 Feb 2014, S.C. Lindstrom, SCL 15627, KP904096
P883, Calvert I., BC, 19 Feb 2014, K. Hind, SCL 15629, KP904097
P884, Calvert I., BC, 19 Feb 2014, K. Hind, SCL 15630, KP904098
P885, Calvert I., BC, 19 Feb 2014, K. Hind, SCL 15631, KP904099
P888, Calvert I., BC, 20 Feb 2014, S.C. Lindstrom, SCL 15635, KP904100

P889, Calvert I., BC, 20 Feb 2014, S.C. Lindstrom, SCL 15635, KP904101
P890, Calvert I., BC, 20 Feb 2014, S.C. Lindstrom, SCL 15635, KP904102
P891, Calvert I., BC, 20 Feb 2014, S.C. Lindstrom, SCL 15635, KP904103
